# Complement Factor H Gene Abnormalities in Haemolytic Uraemic Syndrome: From Point Mutations to Hybrid Gene

**DOI:** 10.1371/journal.pmed.0030432

**Published:** 2006-10-31

**Authors:** Marina Noris, Giuseppe Remuzzi

## Abstract

Noris and Remuzzi discuss a new study showing an association between atypical haemolytic uremic syndrome and a hybrid complement gene,*CFH/CFHL1.*

A typical haemolytic uraemic syndrome (aHUS) is a rare disease involving haemolytic anaemia, thrombocytopenia, and renal failure. There is growing evidence that the disease is associated with defective control of the alternative pathway of complement [1].

Genetic abnormalities in complement regulatory proteins, including complement factor H (CFH), membrane cofactor protein, and complement factor I, have been reported in 30%, 10%, and 5% of patients with aHUS, respectively [2–4]. CFH is a plasma protein produced by the liver that acts as a central regulator in the alternative pathway of complement activation. CFH acts as a cofactor for complement factor I in the inactivation of the central complement protein C3b to form iC3b. CFH also accelerates the decay of the C3 convertase C3bBb of the alternative pathway, and competes with factor B for binding to C3b [5].

## The *CFH* Gene and CFH Protein

The gene encoding CFH is situated, together with genes for five factor H-related proteins (CFHL1–5), within the regulator of complement activation (RCA) gene cluster at chromosome position 1q32 [6,7]. In 1998 a linkage was established between the RCA gene cluster at 1q32 and the disease [8]. In this region there are several large genome duplications, also known as low copy repeats (LCRs), resulting in a high degree of sequence identity between *CFH* and the five *CFHL* genes. By sequence analysis a heterozygous mutation in *CFH* was found in affected patients and obligate carriers within one family, providing the first evidence that CFH genetic abnormalities played a role in aHUS [8].

The secreted protein product of *CFH* consists of 20 repetitive units, named short consensus repeats (SCRs), each composed of 60 amino acids [5]. Around 70 *CFH* mutations have been reported in patients with aHUS (listed in http://www.FH-HUS.org/, The Factor H-Associated HUS

There is growing evidence that the disease is associated with defective control of the alternative pathway of complement.

Mutation Database). Most of these mutations are heterozygous missense mutations that cluster in the exons encoding SCRs 19 and 20. Functional studies on the mutant proteins have shown reduced C3b and heparin binding [1] that results in impaired control of complement activation on the endothelial cell surface. These mutations include two nucleotide changes, c.3572C>T and c.3590T>C, that in some patients occur in combination. As the exon encoding CFH SCR20 differs from the exon encoding CFHL1 SCR5 only at the c.3572 and c.3590 nucleotides [9], the combined mutation makes CFH SCR20 identical to CFHL1 SCR5, a phenomeon known as gene conversion.

## A New Study Showing a Hybrid Complement Gene

Theoretically, LCRs such as those seen in the RCA cluster not only predispose to gene conversion events but are also associated with genomic rearrangements [10], which usually result from nonallelic homologous recombination (i.e., a recombination among different domains and exons with high homology that can generate new gene structures) between LCRs. There is already evidence that a nonallelic recombination between *CFHL1* and *CFH* that results in the deletion of *CFHL1* occurs as a common polymorphism in the general population. In a new study in *PLoS Medicine*, Goodship and colleagues [11] tested the intriguing hypothesis that a particular nonallelic recombination may occur in patients with aHUS that leads to the formation of a hybrid *CFH/CFHL1* gene [11].

To test their hypothesis, the authors studied a large pedigree comprising eight patients over four generations who developed severe forms of aHUS. Although linkage analysis had shown evidence of disease segregation with the RCA cluster [8], a mutation search in two candidate genes, *CFH* and *MCP*, within this region failed to show evidence of any mutation. Now the authors provide strong evidence that the affected and obligate carriers within this family carry a heterozygous hybrid gene that derived from a crossing-over between intron 21 of *CFH* and intron 4 of *CFHL1*. The hybrid gene consists of the first 21 exons of *CFH* (encoding SCRs 1–18 of CFH) and the last two exons of *CFHL1* (encoding SCR4 and 5 of CFHL1). The protein product of the hybrid gene is identical to the CFH mutant protein S1191L/V1197A, which arises by gene conversion (i.e., the aforementioned c.3572C>T and c.3590T>C) and lacks surface complement regulatory activity.

These results provide a new mechanism leading to CFH abnormalities in aHUS and show the complexity of genetic screening of *CFH* family genes. Since the hybrid gene cannot be picked up by genomic screening, which uses *CFH*-specific primers, theoretically some patients that apparently have normal results on *CFH* screening may instead carry a hybrid gene. Based on this hypothesis the authors suggest [11] performing *CFH* screening by multiplex ligation-dependent probe amplification in all patients with aHUS. Their position is strengthened by finding the hybrid gene in three additional unrelated patients with aHUS, indicating that the abnormality is not restricted to a single pedigree. However, larger studies in other independent patient cohorts should be performed to establish the frequency of such an abnormality within the aHUS population.

## Clinical Implications

Identification of the specific genetic defect in patients with aHUS could enhance diagnostic precision, predict clinical outcomes, and, hopefully, translate into improved management of the disease. As CFH is a plasma protein produced mainly by the liver, kidney transplantation in patients with aHUS with *CFH* mutations who have progressed to end-stage renal disease is associated with 80% of graft loss due to disease recurrence [12]. Patients with aHUS should therefore be screened for any possible *CFH* abnormality before inclusion on a transplant list to avoid exposing the patient to an unacceptable risk of disease recurrence. On the other hand, patients with *CFH* abnormalities will benefit from ongoing efforts to obtain specific replacement therapies with plasma fractions enriched in CFH.

## Next Steps

Nowadays, screening for gene mutations is being performed at several clinical and research centres in industrialized countries. However multiplex ligation-dependent probe amplification, which is required to identify the hybrid gene, is a very specialized procedure and few centres may be equipped to perform it. Thus further work is needed to identify and validate an easier test to reliably identify the *CFH/CFHL* hybrid gene. Whether other recombination events could occur in aHUS, generating different *CFH/CFHL* hybrid genes, should also be investigated.

## Abbreviations

aHUS: atypical haemolytic uraemic syndrome
CFH: complement factor H
LCR: low copy repeat
RCA: regulator of complement activation
SCR: short consensus repeat
